# Covalent Organic Frameworks (COFs) as Multi-Target Multifunctional Frameworks

**DOI:** 10.3390/polym15020267

**Published:** 2023-01-04

**Authors:** Syed Nasir Abbas Bukhari, Naveed Ahmed, Muhammad Wahab Amjad, Muhammad Ajaz Hussain, Mervat A. Elsherif, Hasan Ejaz, Nasser H. Alotaibi

**Affiliations:** 1 Department of Pharmaceutical Chemistry, College of Pharmacy, Jouf University, Sakaka 72388, Saudi Arabia; 2 Department of Pharmaceutics, College of Pharmacy, Jouf University, Sakaka 72388, Saudi Arabia; nakahmad@ju.edu.sa; 3 Center for Ultrasound Molecular Imaging and Therapeutics, Pittsburgh Heart, Lung, Blood and Vascular Medicine Institute, University of Pittsburgh, Pittsburgh, PA 15213, USA; amjadm@upmc.edu; 4 Centre for Organic Chemistry, School of Chemistry, University of the Punjab, Lahore 54590, Pakistan; mahussain.chem@pu.edu.pk; 5 Chemistry Department, College of Science, Jouf University, Sakaka 72388, Saudi Arabia; maelsherif@ju.edu.sa; 6 Department of Clinical Laboratory Sciences, College of Applied Medical Sciences, Jouf University, Sakaka 72388, Saudi Arabia; hetariq@ju.edu.sa; 7 Department of Clinical Pharmacy, College of Pharmacy, Jouf University, Sakaka 72388, Saudi Arabia; nhalotaibi@ju.edu.sa

**Keywords:** sonochemical synthesis, photochemical synthesis, dynamic covalent chemistry, stability, crystallinity, anticancer

## Abstract

Covalent organic frameworks (COFs), synthesized from organic monomers, are porous crystalline polymers. Monomers get attached through strong covalent bonds to form 2D and 3D structures. The adjustable pore size, high stability (chemical and thermal), and metal-free nature of COFs make their applications wider. This review article briefly elaborates the synthesis, types, and applications (catalysis, environmental Remediation, sensors) of COFs. Furthermore, the applications of COFs as biomaterials are comprehensively discussed. There are several reported COFs having good results in anti-cancer and anti-bacterial treatments. At the end, some newly reported COFs having anti-viral and wound healing properties are also discussed.

## 1. Introduction

Crystalline porous materials containing light elements connected reversibly in two to three dimensions through covalent bonds is a developing category of Covalent Organic Frameworks (COFs) [1]. Due to unique properties and broad applications, interest in the nanoporous materials field has increased. Scientists used different methods to synthesize porous materials. Before the introduction of the reticular chemistry concept, it was difficult for scientists to prepare discrete porous size organic polymer networks [2]. Metal-organic Frameworks (MOFs) were the first porous materials that were synthesized using the reticular chemistry concept. MOFs were synthesized using polytopic linkers which form a network of polyatomic inorganic metal-containing clusters [3]. The first successful porous organic frameworks are COFs, which are covalent in nature and are capable of integrating organic blocks with precision into a systematic structure [4]. COFs can be prepared by applying the dynamic covalent chemistry concept to stitch organic building blocks together [5,6]. COF crystallization through error correction and self-heating in which structural disorders are repaired dynamically is facilitated by reversible thermodynamic bond formation [7]. COFs have high thermal stabilities, possessing low mass densities with permanent porosity as they are constructed from light-weight elements connected together through strong covalent bonds [8]. Different aspects of COFs have been investigated, reviewed, and reported by a number of researchers [9,10,11,12,13]. COFs are further classified into two categories depending on their building block dimensions:

Two-dimensional (2D) COFs;

Three-dimensional (3D) COFs.

## 2. Synthesis and Main Components

### 2.1. Synthetic Approaches

#### 2.1.1. New Energy Sources

Using different energy resources, COFs can be synthesized. Stable crystalline products which are thermodynamically stable can be synthesized using thermal energy [14]. High energy input and a long time is required for such reactions. Alternative energy resources, such as mechanical agitation, light irradiation, ultrasound, electron beams, and a microwave, can be employed to improve the crystallites nucleation and synthesis of COFs [1].

#### 2.1.2. Microwave-assisted Solvothermal Synthesis

Different functional materials like MOFs [15], metal oxides [16], zeolites, [15] and organic molecules [17] have been synthesized using microwave-assisted reactions. This type of synthesis achieved interest as it provides good reaction attributes, such as low energy consumption, accelerated reaction rates, and higher yields. The first microwave-assisted synthesis was reported by Cooper’s group in 2009 [14]. COF-5 (Boronate ester-linked COF) assembled rapidly through 2,3,6,7,1,11-hexahydroxytriphenlene and 1,4-benzenediboronic acid condensation at 100 °C under microwave irradiation in 20 min. This approach is 200-fold quicker than traditional solvothermal approach [4]. COF-5 possesses a 2019 m^2^g^−1^ Brunauer–Emmett–Teller (BET) surface area greater than the solvothermal synthesized surface area, which is 1590 m^2^g^−1^. Similarly, Boronate ester-linked COF (BTD-COF), which is mesoporous, was synthesized by Bein’s group through microwave-assisted synthesis. Under two sequential heatings for 40 min, a BTD-COF with a 1000 m^2^g^−1^ BET surface area was synthesized, which is highly crystalline [18].

#### 2.1.3. Sonochemical Synthesis

Sonochemical synthesis reduces synthesis time by omitting the induction period. It consumes less energy and is less costly. This synthesis increases the crystallization rate, thus producing high pressures (>1000 bar) and temperatures (>5000 bar) [19]. The first sonochemical synthesis was reported in 2012 by Ahn and co-workers. Under ultrasonication for 1 h, COF-1 and COF-5 (boron-based COFs) were synthesized. COF-5 produced through sonochemical synthesis possess a 2122 m^2^g^−1^ BET surface area and have a higher yield compared to a solvothermal synthesized analog [20].

#### 2.1.4. Mechanochemical Synthesis (MC)

A variety of porous materials have been synthesized through MC synthesis; for example, metal oxides [21], porous carbon [22], MOFs, [23] and graphene derivatives [24]. The synthesis of COFs through MC synthesis is still under development. MC synthesis is ecofriendly, simple, and cost-effective. COF synthesis through solvent-free MC synthesis was first developed by Banerjee’s group in 2013. This was done at room temperature by manually grinding COF precursors for 40 min in a mortar [25]. TpPa-1(MC) and TpPa-2(MC) (COFs) produced via MC synthesis comprised low BET surface area and moderate crystallinity. The COFs showed exceptional behavior in boiling water, 3M NaOH (aq), and 9M HCl (aq) for chemical stability. Another beneficial feature of MC synthesis is the conversion of COFs to 2D nanosheets by spontaneous delamination [26]. A liquid-assisted grinding (LAG) method was used in 2016 by the same group to increase the rate of synthesis of COFs. In the LAG approach, catalytic liquids were added to aggregate the reactants and improve crystallinity and yield. Using this strategy at room temperature, Tp-Bpy-MC (2,2′-bipyridine-based COF) was synthesized by Banerjee and co-workers in 90 min [27].

#### 2.1.5. Photochemical Synthesis

For diversified functional materials, the photochemical synthesis approach has evolved as a beneficial approach. At room temperature and under UV irradiation, the synthesis of COF-5 (UV-COF-5) photochemically was developed by Choi, Lim, and co-workers with a 48-fold increased growth rate and 2027 m^2^g^−1^ BET surface area [28].

#### 2.1.6. Electron Beam Irradiation-induced Synthesis

For the synthesis of functional materials, high-energy radiation was employed. An example of such a synthesis is imine-based COF (EB-COF-1), which results from condensation between 2,4,6-tris-(4-formylphenoxy)-1,3,5-triazine and TAPB under 1.5 MeV electron beam at room temperature. This took about 160 s [29]. This methodology ensures the fast synthesis of COFs and led to pathways for the industrial manufacturing of COFs.

### 2.2. Different Key Components

#### 2.2.1. Dynamic Covalent Chemistry

Kinetically regulated processes that generate irreversible covalent bonds have traditionally dominated the production of polymeric materials. Using irreversible processes, the crystallization of linked organic polymers is difficult. Dynamic covalent chemistry (DCC), conversely, results in the advancement of reversible covalent bonds that can be generated, disrupted, and reformed [30]. DCC is thermodynamically controlled and, unlike traditional covalent bond creation, it results in a reversible reaction that is capable of “proofreading” and “error checking” properties producing structures that are thermodynamically stable. COF synthesis through the DCC concept results in a polymer skeleton which is formed concurrently with the crystallization process. Self-healing feedback lowers structural disorders and aids in the creation of an arranged structure [8]. A highly thermodynamically stable product with a well-defined crystalline structure is produced as a result. In order to maintain thermodynamic control in reversible reactions, involving reaction conditions and reaction media, two main issues must be considered in designing and manufacturing COFs: the structure of the key components and the synthetic method [30].

#### 2.2.2. Structure of Key Components

The structure of the key components must fulfill two basic criteria in order to acquire a crystalline and regulated COF [8]:

(1) The COF formation reaction must not be irreversible reaction;
(2) The shape of key components must be highly conserved in COF.

**Figure 1:**
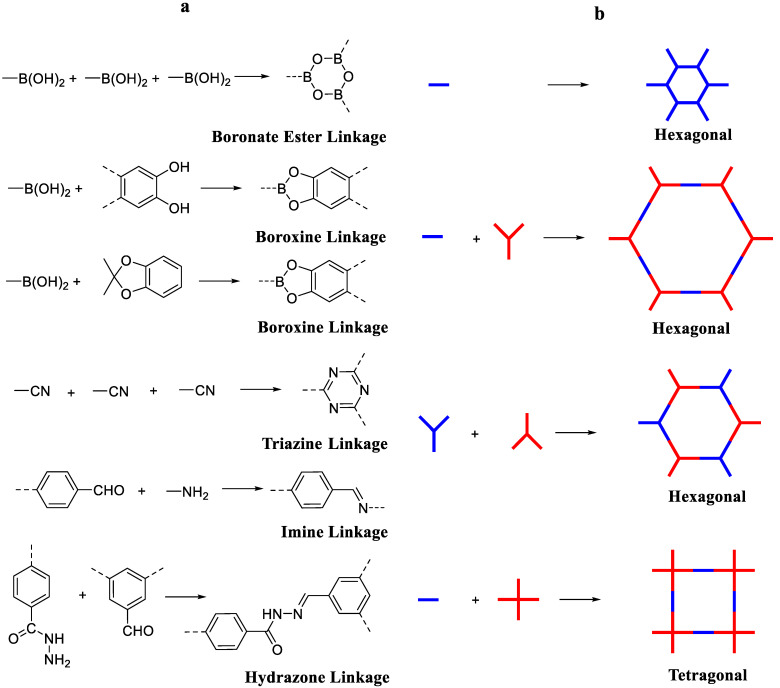
(**a**) Representation of chemical reactions for synthesis of COFs. (**b**) The combination of monomers to design 2D COFs.

The key components must have groups that are reactive enough to activate dynamic covalent bond creation, i.e., there should be no non-reversible side reactions, and under thermodynamic conditions, there should only be oligomers in the reaction system, polymers, and monomers that can be exchanged. Building blocks must be conformational and stiff, and the orientation of bond formation should be distinct. To fulfill the first condition for the successful creation of COF, different reversible processes have been investigated. The chemistry of most of the known structures is boron-based. Boronic acids self-condensate with dialcohols to form five- and six-membered boronate and boroxine ester linkages, respectively [4]. In the presence of the catalyst BF_3_OEt_2_, which is a Lewis acid, a reaction between boronic acids and acetonide-protected catechols took place to generate COFs (Figure 1) [31]. This deprotection approach in situ is a useful way to avoid oxidation and solubility issues with the construction elements, particularly big aromatic dialcohols. Tetraboronic acid condensation with tert-butylsilanetriol is used to create borosilicate linkages, which have been employed to create COFs, similar to boroxine or boronate esters [32].

Despite their heat resistance property, COFs (boron-based) are vulnerable to strike and can react with airborne water vapors [33]. Yaghi’s team invented new COF connection chemistries [34]. Under ionothermal conditions, the cyclical trimerization of cyano groups generated covalent triazine-based frameworks (CTFs) [35]. CTFs have excellent chemical, mechanical, and thermal stabilities, as well as a high-level of conjugation; nevertheless, due to the weak reversibility of the trimerization reaction, they often have low crystallinity [36].

### 2.3. Types of Synthesized COFs 

COF materials have successfully been prepared by using a wide range of building blocks under optimal reaction parameters. The reported COFs are divided into three types based on their structural characteristics [37]:

Boron-containing COFs;

Triazine-based COFs;

Imine-based COFs.

#### 2.3.1. Boron-Containing COFs

**Figure 2:**
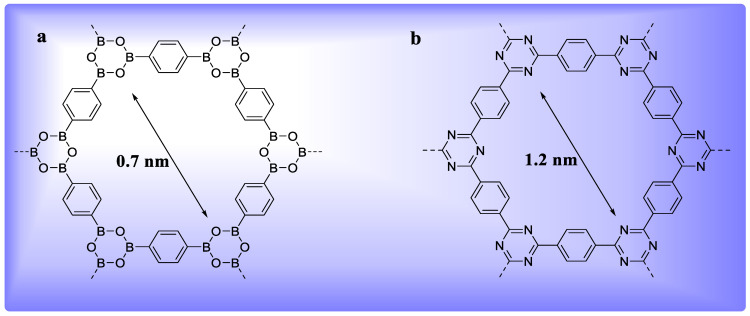
(**a**) Boron containing COF. (**b**) Triazine-based COF (CTF) [37]. “Used with permission of Royal Society of Chemistry, from Covalent organic frameworks (COFs): From design to applications. Ding, S.-Y.; Wang, W., Chem. Soc. Rev. 2013, 42, 548–568 in 2022; permission conveyed through Copyright Clearance Center, Inc.”

Boron-containing COF (Figure 2a) synthesis via boronate anhydride or boronate ester has grabbed researchers’ interests after Yaghi and co-workers’ first creation of COF-1 and COF-5 [4]. Interestingly, the most produced COFs are boron-containing compounds, which could be organized into two groups based on their synthetic techniques. COFs made from the self-condensation of mono building units represents one class of boron-containing COFs. For instance, a 1,4-benzenediboronic acid self-condensation reaction produced 2D COF-1 [4]. COFs made through the co-condensation of two or more building components make up another major group of boron-containing COFs [38]. Boronic acid and diol combine to generate five-membered C_2_O_2_B rings in a dehydration reaction. In case of 2D COFs, where the 2D neighboring layers are mounted vertically, layered eclipsed structures are frequently generated. Three-dimensional COFs with complex architectures can be created using tetrahedrally structured building elements. The benefit of this co-condensation technique is the wide range of boronic acid and diol combinations that may be used as building blocks to create a variety of COFs with various characteristics and functions. A 2D COF material (HHTP-DPB COF) with a large pore size (4.7 nm) was successfully generated through the co-condensation of monomers [39]. Furthermore, this co-condensation technique could be used to adjust the functionality of COF materials. For example, tetra(4-dihydroxyboryl-phenyl)methane 26 and tert-butylsilane triol [t BuSi(OH)_3_] co-condensation produced 3D COF-202 with butyl functional groups via borosilicate formation [32].

Two hydroxyl groups of one –B(OH)_2_ react separately with separate triols, unlike the boronic acid dehydration reaction which generates C_2_O_2_B rings, which are five-membered [40]. It is worth noting that the ^1^HNMR spectroscopic examination of digested COF materials can be used to evaluate the proportion of integrated monomer.

#### 2.3.2. Triazine-Based COFs (CTFs)

Thomas and colleagues created a category of COFs, known as covalent triazine-based frameworks (CTFs) [40]. Nitrile building blocks at 400 °C in the presence of ZnCl_2_ undergoes cyclotrimerization to produce CTFs. The CTF-1 (Figure 2b) material, for example, is made by cyclotrimerizing 1,4-dicyanobenzene (DCB) and possesses a 791 m^2^g^−1^ BET surface area and 1.2 nm pore size. The Powder X-ray diffraction (PXRD) design revealed that CTF-1 has a 2D hexagonal structure like that of COF-1. Surprisingly, a greater ZnCl_2_ (10:1) ratio results in an amorphous polymer with a greater surface area (1123 m^2^g^−1^). The super crystalline CTF-2 material was also prepared successfully using 2,6-dicyanonaphthaline, with a 90 m^2^g BET surface area [36]. The slightly staggered organization of the material layers may account for the reduced surface area of CTF-2. Similarly, increasing the ZnCl_2_ ratios increased the surface area of CTF-2 [41]. Triazine-based COFs (CTFs) have less crystallinity than boron-containing COFs, though they have good chemical and thermal stability. CTFs as catalyst supports have potential usage due to their great nitrogen content [42].

#### 2.3.3. Imine-Based COFs

**Figure 3:**
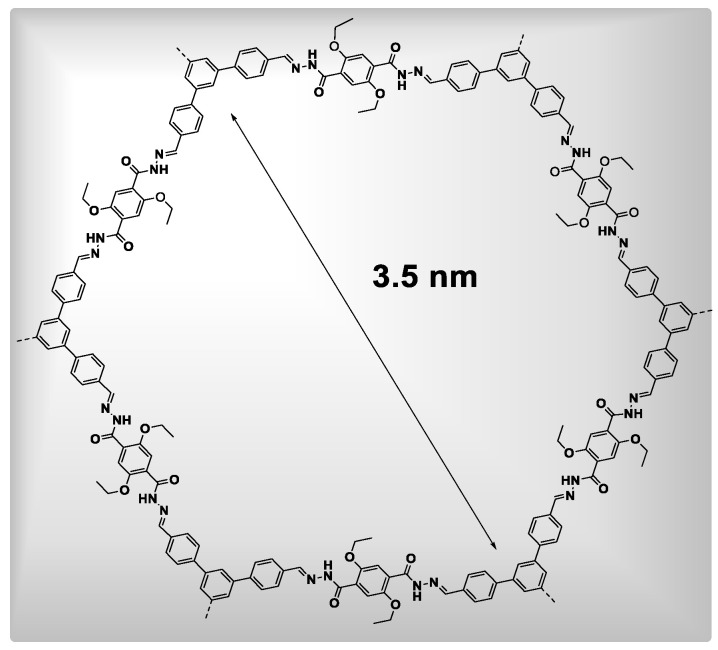
Imine-based COF having pore size of 3.5 nm.

The imine-based COFs, produced by Yaghi and co-workers, represent the third class of COFs [43]. As per covalent production of unique –CQN– bonds, there are two categories of imine-based COF production. One category is “Schiff base,” which is generated via the co-condensation of amines and aldehydes. Yaghi and colleagues created the first imine-based COF in 2009, COF-300 [43], via the dehydration of aldehyde and amine (Figure 3). COF-300 possesses a 3D structure which is like a diamond with a 1360 m^2^g^−1^ BET surface area and 7.8 Å pore size. In 2011, the COF-LZU1 material was created by a co-condensation reaction between 1,4-diaminobenzene and 1,3,5-triformylbenzene for use in catalytic applications [44]. COF-LZU possesses a 2D eclipsed configuration with a 3.7 Å distance among layers, thus allowing metal ions to be included. COF-366 containing porphyrin has recently been prepared and utilized further for optoelectronic applications [45]. Yaghi and co-workers generated hydrazone-linked COFs, which are another form of imine-based COFs, synthesized via hydrazide and aldehyde co-condensation. COF-42 and COF-43 were efficiently synthesized through a solvothermal method using aldehyde and hydrazide as building ingredients [34]. COF-42 and COF-43 are 2D eclipsed structures with BET surface areas of 710 m^2^g^−1^ and 620 m^2^g^−1^, respectively. An abundance of H_2_ bonding present inside hydrazone units aids in the creation of eclipsed structures. Furthermore, the chemical and thermal stability of these hydrazone-linked COFs was excellent [46].

In case of functional consistency, imine-based COFs (CTFs) surpass triazine-based COFs (CTFs), and their crystallinity is comparable to boron-containing COFs. A sequence of metal ions may also be used to coordinate the nitrogen atoms within the framework [47]. These advantages give imine-based COFs much room for improvement in the future, especially for a variety of applications. Amorphous porous polymers relying on the “Schiff base” category were recently introduced, even though there are very few examples of imine-based COFs available [46]. For example, Nguyen and co-workers employed diamine monomers and a 1,3,5-triformylbenzene co-condensation strategy to make various amorphous polymers. However, unlike the synthesis of imine-based COFs, the reaction was carried out under different conditions. This emphasizes the significance of optimizing reaction conditions for COF synthesis and also gives significant data for the development of novel imine-based COFs [37].

## 3. Features of COFs

The atomic layer structure and the conceptual model can be created by managing the geometry, size, and efficacy of the building units. Using proper knot and binder structures in topology diagrams, the qualities of COFs could be settled in this context. COFs created in this way have a unique set of characteristics which are not present in any other material [48].

### 3.1. Low Density 

COFs contain light elements and have excellent gravimetric performance for storing energy and guest molecules. COF-108 comprises 0.17 g cm^−3^ density, which is less than any other crystalline solid [49].

### 3.2. Stability 

When compared to most MOFs, COFs are more stable because they are joined by strong covalent connections. Recent developments (low amine bond polarity, hydrogen bond contacts, keto-enol tautomerization, Michael addition–elimination/benzoxazole mechanism) have reinforced COF structures [50]. These approaches for stabilizing COFs produce materials which are hydrophobic, can withstand a large series of pH, and can be used in both reductive and oxidative conditions that have never been demonstrated before in a MOF.

### 3.3. Crystallinity

COFs are crystalline materials and allow a highly regulated and predictable positioning of functional groups. This describes the structure connection and permits diffraction techniques for characterization. This structural consistency could also be helpful for catalysis and optoelectronic devices [48].

### 3.4. Porosity

COFs possess a regular and systematic permeability, enabling superior functioning in catalysis and gas separation where a complete approach to pores is typically needed. For 2D and 3D COFs, surface areas above 3000 and 5000 m^2^g^−1^ were recorded, respectively [48].

### 3.5. Modularity

Before synthesis, the flexible features of COFs could be altered via the careful selection of building units. As a result, scientists could manipulate the structure and composition of a crystalline and porous material while maintaining a proper hold over mass/volume, chemical functionality, and active site temporal arrangement. COF structures may now be more sophisticatedly regulated with the help of newly discovered tactics, such as integrating monomers with reduced symmetry, multiple bond formation processes, and metal coordination approaches [51]. COFs can be constructed at three structural levels, depending on the required characteristics: skeletal model, design of pores, and complementary model of pores and skeleton [52]. COFs are desirable tenets for directed molecular fabrication that has yet to be completely explored.

## 4. Applications

### 4.1. Catalysis

Wang and colleagues presented the use of COFs for catalysis for the first time in 2011 [44]. Pd/COF-LZU1 was made by the post-metalation of a 2D imine-linked COF and then investigated for the Suzuki–Miyaura coupling process. The catalytic activity of COF was shown to be dependent on the presence of Pd species embedded in the skeleton, which acted in conjunction with the porous nature of the COF. The pure COFs have exhibited catalytic activity for various essential processes during their development, and the use of biomolecular components to create COF-based catalysts has also been investigated. All of these developments show that COFs are potential porous materials for catalysis. This capacity could be due to their inherent catalytic activity and ability to restrict other catalytic elements [53].

#### 4.1.1. Chemical Catalysis

Chemical catalysis plays an important part in chemical manufacturing from an industrial standpoint because of its capacity to increase chemical production and the ease of replication [54]. COFs are extremely promising chemical catalysts because of their high surface areas, high porosity, and high chemical and thermal stabilities. Chemical catalysis has been reported using COFs with reactive skeletons, reactive dangling groups, and reactive metals. Furthermore, the catalytic performance of the developed COF-based chemical catalysts was investigated using a variety of chemical reactions, such as organic coupling reactions, oxidation–reduction reactions, addition reactions, degradation/conversion reactions, and so forth [53].

#### 4.1.2. Photocatalysis

Photocatalysis is a green energy application that uses photons as a source of energy to catalyze reactions within materials having reactive catalytic sites [55]. COFs are good candidates for photocatalysis because they are extremely porous materials with excellent thermal and chemical durability, as well as the capacity to customize the ornamentation of light-harvesting active sites [56]. For example, 2D COFs made up of tabular stacked 2D sheets potentially create highly organized-electron channel systems, allowing charge carrier transport in the stacking direction. Furthermore, COF structures might be designed to have a low band–gap energy, allowing them to absorb photons efficiently across a wide range of light wavelengths. Interestingly, many COFs have displayed amazing optoelectronic features, such as charge carriers, implying that they have a bright future in photocatalysis [57]. Furthermore, COFs’ large surface areas may encourage high gas uptake, making them essential for accelerating gas photoreduction (e.g., CO_2_) [58].

#### 4.1.3. Electrocatalysis

Electrocatalysis is one of the most important concerns for society’s long-term sustainability due to the rising need for renewable energy storage and conversion devices [59]. Heterogeneous electrocatalysis is a technique for speeding up electrochemical processes on electrode surfaces [60]. Moreover, materials used in electrocatalysis must have high conductivity, a large surface area, a large number of catalytic sites, and excellent chemical and thermal stability. Therefore, porous carbons and other 2D materials are widely employed in electrocatalysis applications [61]. COFs are porous materials with excellent structural stability, high porosity, and significant chemical/thermal stabilities, as described in the preceding sections. Furthermore, the design of their components allows for the precise adjustment of their spatial arrangement inside the established structure [62]. This synthetic platform allows for the careful placement of electroactive moieties inside the framework of COFs. Two-dimensional COFs with layer-stacking structures, in particular, accelerate charge carrier mobility via a multi-stacked columnar channel, resulting in fascinating optoelectronic phenomena, electroactivity, and conductivity [45]. COFs, on the other hand, have been intensively researched for proton and electrical conductions, demonstrating their potential as electrocatalyst materials [63].

#### 4.1.4. Organocatalysis

Multifunctional COF polymers offer a lot of potential as economical durable catalysts and have been used in a variety of organic chemical reactions [64]. Integrating nitrogen-containing chemical units into a configurable COF, such as an imine, amine, and triazine group, could help lock metal active sites. As a result, heterogeneous functionalized catalysts based on covalent polymer-carriers have high activity and can be recovered and reused easily [65].

### 4.2. Environmental Remediation

#### 4.2.1. Adsorption

In recent years, COF-based adsorbents have made substantial progress. COFs and derivatives have been used to remove various pollutants from a variety of media. Their use in an adsorption field is explained below [66].

##### Adsorption in Aqueous Solution 

Metal Ion Adsorption

**Figure 4:**
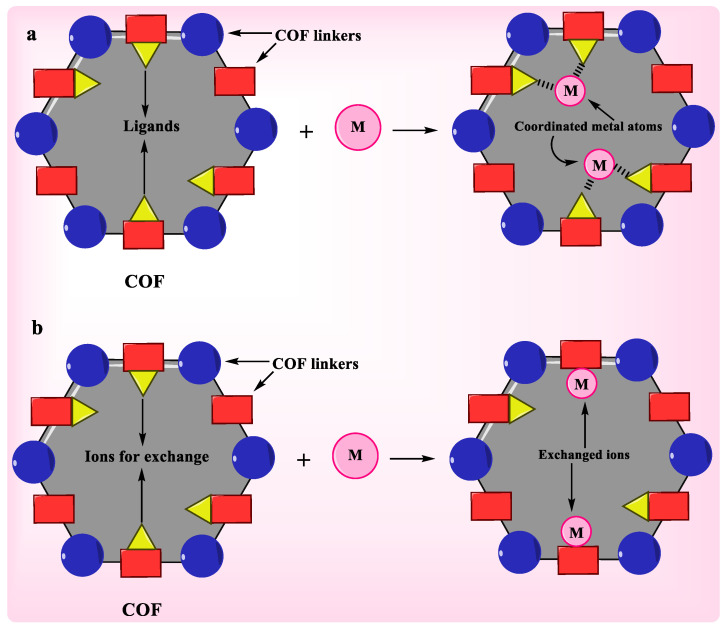
Coordinative adsorption (**a**) and ion-exchange method (**b**) [66].

Heavy metal and radionuclide contamination are a major menace to the ecosystem due to their very harmful effects. As a result, they must be eliminated by required procedures before being discharged into the aquatic environment [67]. COFs’ adsorption-based removal of metal pollutants from water could be a very successful method. COFs with great adsorption aptitude, favorable adsorption kinetics, and selectivity between various ions in water have been regarded for the successful removal of metal ions from water; for instance, Nd^3+^, Hg^2+^, UO_2_^2+^, and TcO^4−^ ions. Coordinative adsorption and ion exchange via ligand domains on the surface of COFs is the most common adsorption process [68]. Figure 4 depicts coordinative adsorption and the ion-exchange method [66].

Adsorption of Organic Contaminants

**Figure 5:**
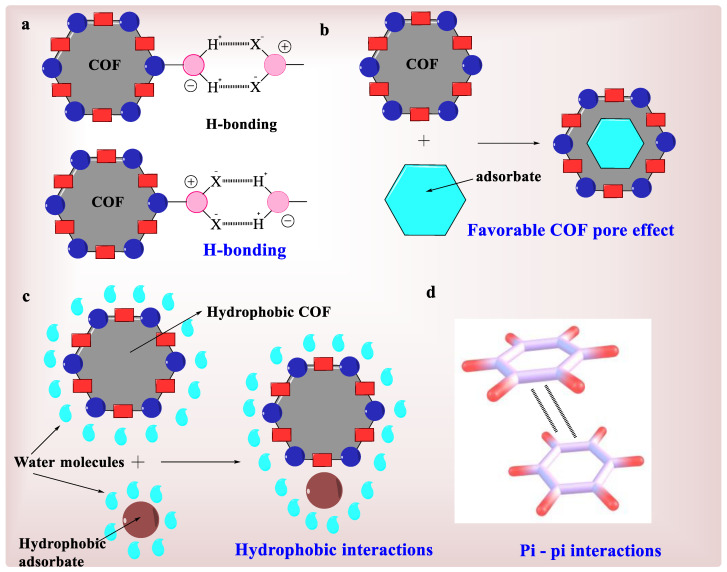
Major adsorption mechanisms; H-bonding (**a**), pore-size effect (**b**), hydrophobic interaction (**c**), and pi–pi interactions (**d**).

The widespread usage of pesticides, coloring agents, and PPCPs is a critical disquiet because they contaminate water and the majority of them are organic chemicals [69]. They can be hazardous to flora and fauna, and they can disrupt ecosystems by disrupting the oxygen/nitrogen balance in the water. They can spread and pollute a large region far beyond their point of origin through water flow. In many cases, only a trace amount of the contaminant is required to produce significant environmental and human health issues [70]. So, organic pollutants should be removed as much as possible before being discharged into a water system. Because of their broad, rigid, and persistent permeability, as well as appropriate surface FGs, COFs looked promising for massive organic compounds adsorption. Different adsorption methods have been observed depending on the impurities and the nature of COF functioning. Pore-size effect [71], H-bonding [72], hydrophobic interaction [68], and p–p interaction [73] are all major adsorption mechanisms, as shown in Figure 5. As evidenced by the capture of arylorganophosphorus flame retardants [74], pore size effects may sometimes become a significant cause in adsorptive removal.

##### Gas–Phase Adsorption

COF-based materials have not been extensively employed for gas adsorption, even though their potential in this field is tremendous. COFs and similar materials have primarily been used for CO_2_ capture. COFs have also effectively adsorbed various gases/vapors, such as I_2_ and CH_3_I. These will be described further below.

CO_2_ Capture

One of the most important environmental issues of the twenty-first century is CO_2_ buildup in the atmosphere. Removing CO_2_ from the atmosphere or capturing flue gases before they are released into the atmosphere are crucial research subjects [75]. COFs are shown to be excellent materials for specific CO_2_ adsorption from a mixture of gases. The concept of employing COFs as CO_2_ adsorbents was first proposed in a theoretical study [76]. COF use for capturing CO_2_ was first published in 2008 [77]. Several results for CO_2_ adsorption were then published in a short time. COFs based on triazine, imine, boron, and boron/imine have been used to entrap CO_2_ thus far [78].

#### 4.2.2. Filtration/Separation 

COF-oriented membranes are still in their early phases of usage for separation and filtration, such as gas adsorption. COFs are frequently obtained as powders, which are incompatible with filtration and separation membranes. As a result, COFs constructed with polymer matrices could be used to make membranes [79]. COFs can also be grown on support materials to make membranes. COF-based membranes’ separation performance can be impaired by the masking effect of polymers, poor support adhesion, and poor crystal formation [80]. The compatibility of interface with a polyvinyl alcohol matrix could be improved by separating COFs into nanosheets by intercalating n-BuLi [81]. COF membranes are used in many applications, including water treatment and gas separation.

##### Gas Separation

Gas separation has also been accomplished using COF-based membranes. As constituents in mixed-matrix membranes, a variety of COFs with varied pore apertures and covalent connections was studied. COFs as fillers have been utilized in these membranes, and they function in the same way that they do in polymer matrices. For this, the persistent matrix phase polymer and dispersed filler phase must have similar strong affinity [66]. In these membranes, a synergistic effect has been observed among COFs and the polymer matrix, resulting in enhanced composite absorption. The amount of COF that is loaded into the mixed-matrix membrane has a significant impact on extraction efficiency. Due to the sheer role of well-spaced-out channels, low COF impregnation improves separation performance. However, if there is moderate COF in the membrane, the gas transport channels can become clogged, resulting in poor performance. For example, separating membranes with 0.4 wt% of COF-5 had the best CO_2_/N_2_ selectivity and CO_2_ permeability when matched with other COF-5/polyether block amides (Pebax-1657 matrix), membranes which contain different COF loadings [69].

### 4.3. Sensors Based on COFs

Certain luminous COFs can modify their luminescence properties (whether augmenting, dampening, or switching) in the presence of specific analytes. As a result, analytes can be spectroscopically detected or can be seen with the human eye [82]. The usage of COFs as chemical sensors is based on this core idea. Although, such activity does not remove impurity directly; therefore, efficacious sensing or the detection of impurity is required before its withdrawal. Metal ions [83], ammonia [84], antibiotics [85], and a range of other chemical substances [86] have all been detected using luminous COFs. In 2016, Ding et al. [82] published investigations to detect Hg^2+^ ions in water by employing a hydrazone-linked COF (COF-LZU8) with thioether groups. The fluorescence was suppressed by transferring p-electrons from the skeleton to Hg^2+^ empty orbitals. As a result, COF-LZU8 could detect this ion at very low concentrations (25 ppb Hg^2+^). Using a similar luminescence quenching technique, a luminous azine-linked COF (COF-JLU3) was employed to sense Cu^2+^ ions [83]. A hydrazone-linked 2D COF [87] detected F^-^ anions at very low concentrations (50.5 ppb) in solution. The deprotonation of N–H by F^-^ resulted in fluorescence amplification in this case. This was interpreted as an acid–base interaction in which F^-^ ions deprotonated the COF surface, resulting in N^-^. Acid–base interactions were used to identify ammonia from the dampening of a boronate-linked COF [84]. Boronate worked as an electron acceptor, while ammonia served as an electron pair donor. Electrochemical impedance spectroscopy was used to detect trace quantities of the antibiotics (enrofloxacin and ampicillin) by using an aptamer-immobilized Py-M-COF [85]. This approach could detect enrofloxacin and ampicillin at low concentrations. Hg^2+^ may be detected with the naked eye using fluorescent N-doped carbon dots and RB@COF composites [88]. The composite’s fluorescence emission was reduced at 440 nm and elevated at 570 nm in the presence of Hg^2+^. In conclusion, considering the enormous potential and the sheer lack of study, the application of COFs as sensors shows significant potential [66].

### 4.4. COFs as Biomaterials

Biocompatibility and biofunctionality are the most important characteristics of biomaterials [89]. Other characteristics, for instance, strong corrosion resistance, high wearing resistance, capacity to withstand host responses, and long life, must be evaluated according to the particular application [89]. Metallic biomaterials can be corroded; therefore, the life of medical devices can become reduced, which must be avoided. As a result, biomaterial design and selection are critical for selective application. COFs, such as polymeric biomaterials, are highly designable and functionalizable [90]. Notably, the application of COFs as biomaterials is under development; thus, it is critical to identify areas where COFs could be beneficial.

#### 4.4.1. Drug Delivery

Medications for minor-molecule chemotherapy includes doxorubicin (DOX), cisplatin, and paclitaxel, and have inadequate tumor targeting capability and more adverse effects than larger-molecule chemotherapy agents [91]. Nano-crystallization by nanocarriers can efficiently overcome these flaws. Because of improved permeability and retention (EPR) effect, nanosized molecular medicines can accumulate more effectively in tumors [92]. As a result, adverse effects could be considerably decreased, while therapeutic outcomes could be greatly improved.

COFs include built-in and adjustable porosity channels that allow medicinal molecules to be loaded. For example, drug entrapment quantity and COF release rate could be adjusted in a given series by systematically controlling pore size and polarity to fulfill various purposes. In 2015, Yan et al. reported virtual 3D COF (PI-COF-5 and PI-COF-5) models with varying diameters of aperture for drug delivery systems [93]. The ibuprofen release rate was lower in PI-COF-5 with small pores than in PI-COF-4. In 2016, Zhao et al. researched captopril, ibuprofen, and 5-fluorouracil (5-FU)-loaded PI-2-COF and PI-3-COF nanomedical systems and investigated cancer cell (MCF-7) suppression using 5-FU@PI-2-COF and 5-FU@PI-3-COF [94].

**Figure 6:**
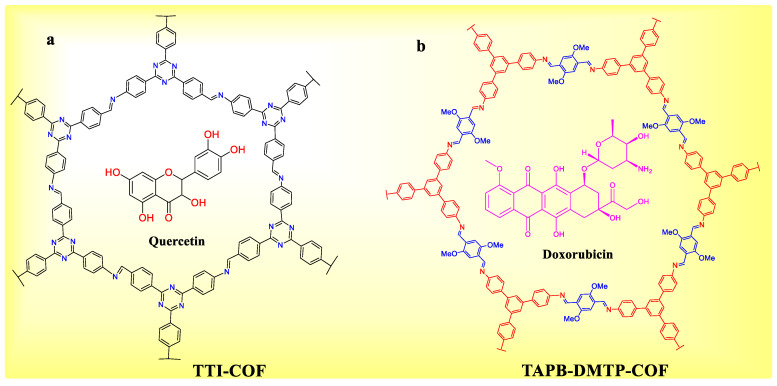
(**a**) Quercetin adsorbed on TTI-COF (**b**) Doxorubicin adsorbed on TAPB-DMTP-COF.

In 2016, Lotsch et al. published innovative research on COF-based intracellular medication delivery [95]. Persistent imine-connected COF (TTI-COF) (Figure 6a) with available electron pairs is bounded reversibly with quercetin guest molecules via non-covalent contacts. TTI-COF without drugs was not hazardous to cells; however, quercetin-loaded TTI-COF prohibited the growth of cells more efficiently than molecular quercetin. COFs are potential drug delivery vehicles, but their µm particle size, low hydrophilicity, and lack of hitting ability severely lower their cellular absorption efficacy, in vitro inhibitory impact, and in vivo applicability [96]. Three strategies, however, can be used to efficiently correct these flaws.

PSM is used to decorate selected groups on COF surfaces in the first technique. In 2017, Banerjee et al. published 5-FU@TpASH-FA derived from TpASH by using three PSM steps [97]. Folate-decorated TpASH-FA primarily transported 5-FU to folate receptor overexpressed MDA-MB-231 cancer cells via receptor-mediated endocytosis, resulting in more efficient necrosis than pristine TpASH. To boost their hydrophilicity, the COF surface is coated with surfactants in the second approach. Jia et al. published a study in 2018 that described COF-1 coated with water-dispersible polyethylene glycol (PEG) as a sophisticated transporter for in vivo medication administration [98]. COF-based nanocomposite PEG-CCM@APTESCOF-1@DOX was made from curcumin-modified PEG (PEG-CCM) and DOX-loaded amine-functionalized COF-1 (APTES-COF-1@DOX). After intravenous administration, the produced PEGCCM@APTES-COF-1@DOX effectively assembled in tumors, resulting in a stronger anticancer impact than the free DOX formulation. It was the first reported in vivo tumor therapy with COF-based medication system [91].

The third technique creates COF nanosheets or nanoparticles (e.g., 5-FU@TpASH-FA). A Schiff base condensation between 2,5-dimethoxyterephthaldehyde (DMTP) and 1,3,5-tris (4-aminophenyl)benzene produced TAPB-DMTP-COF particles (Figure 6b) with 200 nm size [38]. DOX@COF showed admirable in situ antitumor effects when the NCOF was used as a delivery vehicle for DOX due to improved dispersion and cell affinity [99].

#### 4.4.2. Photodynamic Therapy

PDT is microsurgical therapy in which photosensitizers (PSs) capture photons and convert oxygen (O_2_) to reactive oxygen species (ROS; for instance, ^1^O_2_, °OH, and °O_2−_), causing cancer cell destruction [100]. Traditional PSs (such as porphyrin BODIPY) have a low cell absorption due to their weak water solubility, which limits their PDT efficacy. The loading of PSs on nanocarriers, as reported in prior studies, can control these shortcomings and thereby improve anticancer therapy [101].

**Figure 7:**
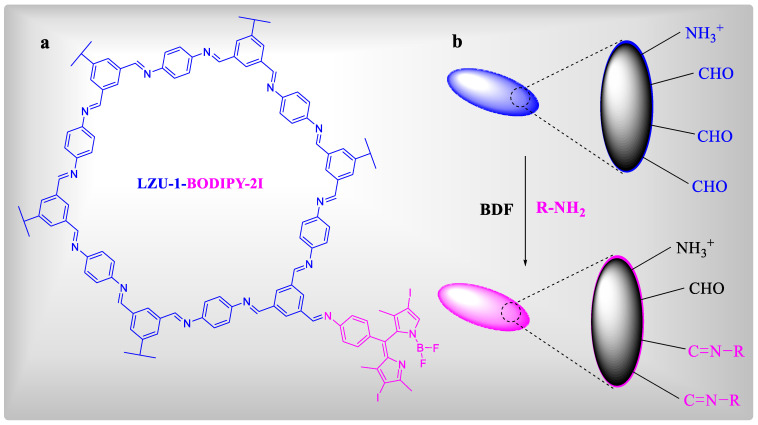
(**a**) COF LZU-1 with a particle size of 110 nm. (**b**) BODIPY PS effectively nanosized on LZU-1 nanoparticles [101].

Dong et al., in 2019, published the first in vitro and in vivo example of NCOF-based PDT [101]. COF LZU-1 (Figure 7a) with 110 nm particle size was made utilizing benzene-1,3,5-tricarbaldehyde and tert-butyl (4-aminophenyl) carbamate as monomers in a polymer-assisted solvothermal process. The BODIPY PS was effectively nanosized on LZU-1 nanoparticles by employing monoamino-decorated BODIPY and a free end aldehyde group via a bonding defect functionalization (BDF) technique, as illustrated in (Figure 7b). Even though the BODIPY concentration was relatively small, the LZU-1-BODIPY2I strongly decreased HeLa and MCF-7 cell survival in green LED illumination (Figure 5c). In comparison to other popular nanomaterials, LZU-1 demonstrated the least hindrance toward MCF-10A normal cells (Figure 5d), showing that COFs may have greater biocompatibility and appropriateness for biomedical applications. Heavy metal ion poisoning is ubiquitous, hence the favorable qualities and toxicity of a wide range of metal-involved nanomaterials are constantly linked [102]. This COF-based work demonstrates that metal-free COFs may be a viable approach to decrease the toxic effects of metal-involved nanomaterials, particularly those containing heavy metals.

The synthesis of photosensitive COFs from ROS inert monomers was recently discovered by Deng et al. [103]. Qu et al. created porphyrin-COF nanodots for tumor PDT (TAPT-DHTA-COF) [104]. The COF nanodots were provided with good ROS generation capability under 638 nm radiation due to well-isolated porphyrin molecules in the skeleton, resulting in exceptional PDT effectiveness in vivo and in vitro. COF nanodots, due to their nano-size (3–4 nm), can be removed through renal filtration from the body without producing enduring toxicity.

Tan et al. grew porphyrin-based COFs on up-conversion nanoparticle surfaces via core-mediated imine polymerization in 2020. (UCNP) [105]. When exposed to near-infrared (NIR) light, the porphyrin photosensitizer of UCNP@COF was excited by red light (654 nm) from UCNP to produce ^1^O_2_. Because NIR light penetrates deeper into the epidermis, UCNP@COF has a stronger antitumor activity in vivo [105].

#### 4.4.3. Photothermal Therapy

**Figure 8:**
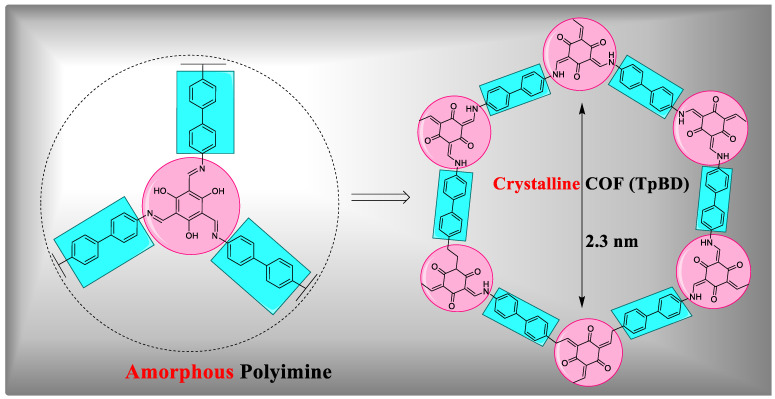
Basic unit (amorphous polyimine) and crystalline COF (Tpbd) “Reproduced with permission of John Wiley and Sons, Angewandte Chemie International Edition from Manipulation of amorphous-to-crystalline transformation: Towards the construction of covalent organic framework hybrid microspheres with NIR photothermal conversion ability by Tan, J.; Namuangruk, S.; Kong, W.; Kungwan, N.; Guo, J.; Wang, C. in Angew. Chem. Int. Ed. 2016, 55, 13979–13984 [108]”.

PTT is a new type of light-based tumor treatment. By using a photothermal agent, laser energy can be converted into heat under near-infrared laser irradiation, thereby ablating the tumor. PTT is a congenital cancer treatment that is frequently employed and is independent of oxygen [106]. COFs may be applied as modifiers to enhance the photothermal effect of nanomaterials or as transporters to distribute photothermal agents in photothermal therapy. COFs may swiftly transmit temperature, in contrast to closed porous structures which slow heat transfer [107]. The possibility of nano-COFs for photothermal therapy was originally assessed by Wang’s team. Fe_3_O_4_@COF(TpBD) with a fundamental shell structure was constructed using Fe_3_O_4_ as a template and modified with polyethylene glycol to increase system stability in solution (Figure 8). According to the characterization data, the specific surface area of TpBD was 1346 m^2^/g, the size of pore was 1.3 to 2.0 nm, and the shell width was 100 nm. The photothermal conversion efficacy of TpBD incorporating COFs was 21.5% at 785 nm, which was two to three times greater than Fe_3_O_4_ nanoclusters alone, according to the results [108]. COFs can be used as photothermal agents. COF cationic radicals exhibit good photophysical properties due to charge transfer among linked multilayers. As a result, Guo’s research group created Py-Bpy + •-COF/PEG, which have photothermal characteristics based on this property. Under 808 nm and 1064 nm and laser radiation, the system’s photothermal conversion efficacy was as high as 63.8 percent and 55.2 percent, respectively [109].

#### 4.4.4. Combined Therapy

Monotherapy due to the complexity and heterogeneity of tumors [110] has several limitations which make the removal of tumors tough. Combined therapy, on the other hand, can effectively drub the drawbacks of monotherapy and boost the curative benefit even more. COFs’ adaptability makes them simple to mix multiple therapies on COF nanoplatforms. In 2019, Chen et al. created COF TP-Por via a combination of 5,15-bis(4-boronophenyl)porphyrin and 2,3,6,7,10,11-hexahydroxytriphenylene [111]. By employing cyanine-assisted exfoliation, TP-Por was able to accomplish nanocrystallization; after additional loading of DOX prodrug, the resulting composite material was able to suppress 4T1 tumor cells successfully by using combined therapy under 808 nm irradiation. Impressively, the produced 2D covalent organic nanosheets (CONs) were employed for the treatment of HeLa tumor via intravenous injection with PDT and PTT under 635 nm radiation after the ultrasonic exfoliation of TP-Por (Figure 8) [112]. Theoretically, CONs’ molecular heterostructure allows efficient charge carrier separation, resulting in long electron and hole lifetimes. Then, the reduction of O_2_ took place to made CO_2_, and the oxidation of H_2_O by holes occurred to produce COH. Meanwhile, heat energy was emitted by non-radiative diminution to acquire PTT. For PAI-guided PDT and PTT, NCOF COF-366 with 5,10,15,20-tetrakis(4-aminophenyl)porphyrin and terephthalaldehyde as monomers were also employed [113].

NCOF therapeutic drugs designed upon host–guest systems were also investigated by Dong et al. VONc@COF-Por, a COF-based dual-modal PDT/PTT therapeutic nanoagent, was created using BDF and guest encapsulation procedures stepwise on NCOF with diameters of around 140 nm. In the presence of red LED and 808 nm laser light, VONc@COF-Por showed greater ^1^O_2_ synthesis and photothermal conversion capacity (55.9%), which irreversibly caused damage to tumor cells’ mitochondria and lysosomes, finally leading to cell death. In vitro and in vivo experiments revealed the strong antitumor efficiency that resulted from both PDT and PTT. Yuan et al. recently used a COF-based theranostic nanoplatform to achieve immunogenic phototherapy [114]. After near infrared PTA indocyanine green (ICG) loading, ultrasonic exfoliation, polydopamine (PDA), and PEG coating, the obtained ICG@COF-1@PDA exhibited ROS generation and regional hyperthermia when exposed to an 808 nm laser. This combination of PDT and PTT caused evident immunogenic cell death in colorectal cancer patients and resulted in untreated distant tumor inhibition. ICG@COF-1@PDA was also found to be effective in treating pulmonary metastases from murine breast cancer [91].

#### 4.4.5. Drug Chemotherapy

COFs possess high loading capability due to their systematic pore structure and huge specific surface area. COFs may sustain steady structure throughout the process. As a result, COFs are employed as transporters in drug chemotherapy to distribute medications to target [115]. Zhao’s group prepared PI-3-COF and PI-2-COF (imine 2D COFs) in 2017 and investigated their drug-loading actions [94]. 2DCOFs have strong biocompatibility and can sustain a good structure of pores in water and present as nanoparticles, according to studies. The drug-loading and ejection of COFs were studied using 5-fluorouracil (5-FU), captopril, and ibuprofen (IBU). PXRD, thermogravimetric analysis (TGA), Fourier-transform infrared spectroscopy (FT-IR), and TGA were used for investigating drug-loading effect of COFs. The MCF-7 cells’ survival rate with a drug-loading system was shown to be significantly lower in the experiments [94].

A simple COF drug-delivery device, like other carriers, does not target cancer cells. In 2017, COFs (TpASH and TpAPH) were created by Banerjee’s team. For targeting (CONs), they improved TpASH and created folic acid-conjugated organic nanoparticles [97]. Through receptor-mediated endocytosis, the selected CONs delivered the medication 5-FU to cancer cells in breasts and destroyed them. The drug-loading device loaded 12 percent 5-FU according to UV-vis analysis. MTT assays and fluorescence microscopy were used to investigate cell migration and absorption, and the results revealed that the system had effective cancer cell targeting. The effective localization of cancer cells in breasts has considerable capability for the delivery of drugs despite the minimal drug loading [115].

However, in vivo COFs that have not been changed exhibit a low water dissolution rate. In 2018, a drug-delivery method for COF-1 was published (Figure 8) [98]. COF nanocomposites with water miscibility by combining PEG-CCM and APTES-COF-1 were created, and a doxorubicin (DOX)-loaded COF drug-delivery system was established. With APTES-COF-1 as the oily phase and PEG-CCM as the surfactant, the system can be thought of as micelles. After reaching the interior of cells, PEG-CCM slips off, releasing DOX. PEG-CCM was used to change COF surfaces, which considerably increased the hydrolysis stability, drug loading, and drug entrapment effectiveness.

**Figure 9:**
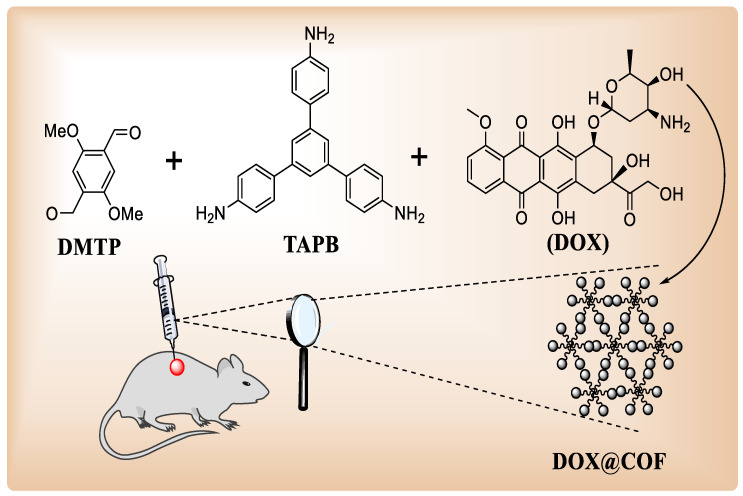
Combining DOX and TAPB-DMTP-COF. “Reproduced with permission of John Wiley and Sons (Chemistry—A European Journal) from One-Pot Synthesis of DOX@ Covalent Organic Framework with Enhanced Chemotherapeutic Efficacy by Liu, S.; Hu, C.; Liu, Y.; Zhao, X.; Pang, M.; Lin, J. in Chem. A Eur. J. 2019, 25, 4315–4319” [99].

Lin’s team used a one-pot approach for assembling COFs and pharmaceuticals in the COF drug-loading system, combining DOX and TAPB-DMTP-COF (Figure 9) [99]. DOX and DMTP were assorted and agitated for 1 h, then it was supplemented with TAPB reagent and a Schiff base reaction was used to form DOX@COF. COFs binding with DOX is good, according to FT-IR measurements. The drug loading of the system was approximately 32.1 percent by weight, substantially greater than previously published data based on DOX absorption intensity in UV/vis spectrum. In addition, mice were given intratumoral injections in vivo, and the results revealed that the method had strong destructive effects on tumors [115].

#### 4.4.6. COFs for Protein Intracellular Delivery

Protein therapy is a contentious issue in biomedical research. Protein medicine, in contrast to traditional small-molecule treatments, have good targeting, appropriate protection, and substantial efficiency. However, approximately 70% of the genome-encoded proteins faced trouble passing across the cell membrane, resulting in significant challenges for creating protein medicines and discovering new protein activities [116]. COFs’ special features, e.g., their high surface area, nontoxicity, tunable architecture, and lipid solubility, enabled scientists to address this issue. The grafting of protein molecules on COFs and then the attachment of polymers of high biological affinity to COFs’ surfaces results in the unique hybrid delivery method for efficient protein delivery within the cells. The protein delivery technique has the advantage of having no chemical modifications of protein and no denaturation of the protein during delivery [115]. It can keep proteins biologically active after they have been delivered.

#### 4.4.7. COFs for Imaging and Diagnosis

##### In Vitro Diagnosis

For better cancer therapy, the diagnosis of cancer in early stages is important. To enhance patient survival rates, early detection methods for cancer must be developed. COFs have a layered structure, great permeability, small density, and excellent biocompatibility and durability. Due to these qualities, they served as excellent transporters for agents to detect in vitro tumor, thus ensuring sensitivity [117]. COFs with a porous structure could interact with a variety of guest molecules at the same time, thus enhancing the detection limit. COFs have many distinct features that help in tumor detection. Liu et al. designed a new aptasensor that uses porphyrin COF (p-COF) to immobilize epidermal growth factor receptor (EGFR)-targeting aptamer strands that can effectively and specifically bind to EGFR to identify cancer cell lines in breasts (MCF-7 cells), based on electrochemical measurements [118]. This p-COF-based aptasensor for EGFR has 0.54 fg mL^–1^, which is a low detection limit (LOD). For MCF-7 cells, the aptasensor based on p-COF had a LOD of 61 cell mL^–1^. The following factors were responsible for the high sensitivity and selectivity [117]:

(1) COFs’ high conjugation not only boosts electrochemical activity but also strengthens the interface between COFs and biological molecules;
(2) The mesoporous channel (2.06 nm) of COFs proved advantageous for aptamer fixation and feeding;
(3) More aptamers could be coupled due to the planar structure of 2D COFs. As a result, the proposed sensor could be employed as a new biosensor platform for instantly and sensitively identifying cancer moieties.

The colorimetric analysis offers particular benefits in quick cancer diagnosis and point-of-care testing (POCT) because of its low cost, simplicity, and great sensitivity [119]. COFs also have chemical flexibility, great porosity, systematic structural integrity, and many binding sites. As a result, COFs are proving to be excellent transporters for a variety of guest molecules. Wang et al. created a water-based stable carboxymethyl cellulose-modified COF hydrogel (Pd NPs/CMC-COF-LZU1) to allow the in situ growth of palladium NPs, which are based upon the principle of colorimetric identification [120]. Folate receptor (FR)-positive cancer cells were targeted via folic acid (FA)-modified Pd NPs/CMC-COF-LZU1, which catalyzed NNPH to NPH, resulting in a change in color and multicolor imaging. This detection technique has a low detection limit and can detect cancer cells in samples (100 cells per milliliter). COFs can efficiently load fluorescent dyes due to their porous nature, with no quenching. However, simultaneously, the skeleton of COFs shields dyes for excellent photochemical stability. The skeleton of COFs also has great anti-interference properties, which make it a suitable fluorescence sensor [121].

##### In Vivo Diagnostic

COFs with enormous permeability, high electrical conductivity, and distinct interactions with bioactive compounds looked very promising in cancer diagnostics (bio-sensing and bio-imaging). Wang et al. employed core-mediated imine polymerization to coat nanoscale COFs (UCCOFs) with varied shell thicknesses by employing a lanthanide-doped upconversion nanoparticle (UCNP) center [105]. The UCCOFs were able to produce singlet oxygen for PDT and radiate singlet oxygen-correlated fluorescence through near-infrared luminescence imaging to monitor the treatment process. The loaded ICG, which was used as an ROS indicator, was gradually degraded by ^1^O_2_. As a result, the UCCOFs-1 luminescence at 800 nm was activated, which located the site and dose of generated ^1^O_2_ in the body, enabling an in situ self-reporting PDT.

Liu et al. designed a pH-responsive nanoplatform based on zinc porphyrin COF (ZnCOF), with a 22.5 percent loading rate by weight of zinc porphyrin (ZnPor) [108]. At pH 7.4, there was no fluorescence signal in the linked ZnPor in the assembled state. The distributed ZnPor showed clear fluorescence signal recovery upon pH-triggered disintegration of ZnCOF in TME at pH = 5.5. Simultaneously, the metal-enhanced fluorescence effect amplified the fluorescence signal of the shed bovine serum albumin (BSA)-coated gold nanoparticles, producing three times more fluorescence in vivo than the ZnPor free group. COF may thus intermingle with imaging molecules to attain the goal of imaging as an excellent carrier. Simultaneously, the development of the COF structure can integrate building blocks with fluorescence qualities directly, resulting in a COF with fluorescence imaging capabilities, in particular, setting. Alternatively, after the establishment of COF, it could achieve fluorescence activation for inactive building blocks [122].

In cancer biosensing and bioimaging, nanoscale COFs possess the following advantages [117]:

(1) Rich π-conjugation is favorable to electrochemical signals via π–π stacking with specific ligands, efficient biological molecules, and so forth;
(2) COF design can provide the optimal particle size for bioavailability;
(3) Functional biomolecules, such as aptamer, can be fixed within COFs with high permeability, enhancing the probe’s adsorption capacity and sensing response;
(4) COFs contain many sites that can bind fluorescent dyes;
(5) COFs made of fluorescent molecules possess biological imaging capability.

Simultaneously, the development of COF structure can stimulate the fluorescence activity of building block.

#### 4.4.8. Covalent Organic Frameworks (COFs) for Enzyme Immobilization

**Figure 10:**
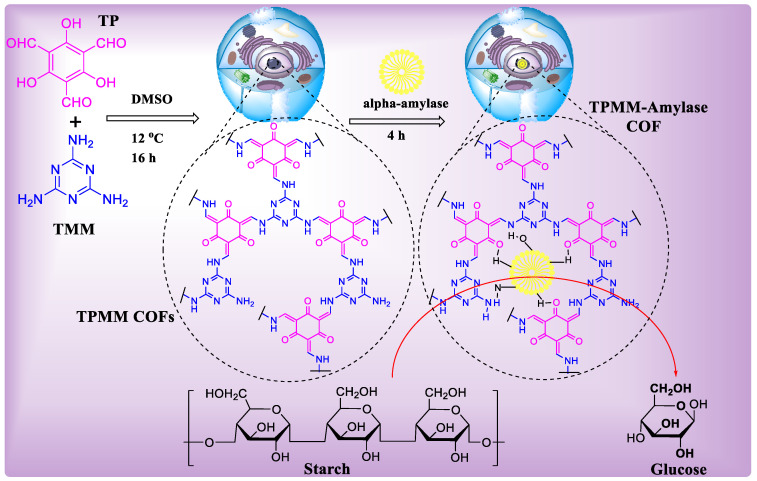
Physical adsorption of a-amylase into the TPMM COF pores “Reprinted from Integration of α-amylase into covalent organic framework for highly efficient biocatalyst, Samui, A.; Sahu, S.K. Microporous and Mesoporous Materials 2020, 291, 109700.” Copyright (2022), with permission from Elsevier [126].

COFs are great candidates for the immobilization of enzymes. COFs for immobilizing enzymes avoid hazardous ion leaching and long-term water/chemical stability issues that MOFs often experience [123]. Numerous studies showed that COFs as enzyme immobilization matrixes can retain enzyme activity and boost operational stability and recyclability [124]. Kandambeth et al., for example, used a template-free technique to synthesize hollow sphere-shaped COFs which have a mesoporous structure in a single step [124]. These COFs were used to immobilize trypsin, which had a high absorption quantity and was crystallizable and chemically stable. Sun’s group carefully investigated the consequence of COF pore environment on the functioning and stability of immobilized enzymes [125]. COFs’ unique and changeable pore shape improved the compatibility of the microenvironment with enzymes, made enzymes more receptive to reagents, and inhibited enzyme inactivation. A novel COF, COF-OMe was created using a mixture of 1,3,5-tris (4-aminophenyl)-benzene and dimethoxyterephthaldehyde (DMTP) in the TPB scheme. The lipase was then infused into COF pores and physically adsorbed to the COF surface [126]. Further research revealed that the alignment of active sites of the enzyme could be controlled by an interaction between both COF components, leading to improved enzyme-COF composite activity. Samui et al. used physical adsorption to immobilize Alpha-amylase (a-amylase) into the TPMM COF pores (Figure 10) [126]. The enzyme-COF composites’ Km values revealed that a-amylase had a high affinity for the substrate. By using GA as cross-linking agent, Qian et al. immobilized trypsin via covalent interaction amid amino and carboxyl groups [127]. Through the deposition of COF (TpPa1) to magnetic graphene (MG@TpPa-1), they were able to design novel enzyme support with high enzyme loading. Further research evaluated the efficacy of free and immobilized trypsin in digesting standard proteins. Furthermore, the BSA sequence area obtained was greater than that obtained using silica biocatalyst coated with cellulose [128], a magnetic graphene oxide trypsin reactor [129], and a multipoint fixed MOF bioreactor [130]. The constructed trypsin bioreactor greatly improved digestion efficiency.

Oliveira et al. used COF (PPF-2) and three different strategies to immobilize Candida Antarctica lipase B (CAL-B): (1) physisorption; (2) covalent bonding by the amino group in N-terminal residue of protein and free aldehyde groups of PPF-2-CHO; (3) covalent connection by the epoxide group and free amino groups of PPF-2-NH2 [131]. CAL-B supplemented with PPF-2 demonstrated high specific activity in oleic acid esterification with ethanol, ranging from 58 to 283 U/mg, which was 2.6 to 12.7 times higher than Novozyme. In 2019, Song’s team used an ammonia–aldehyde condensation procedure to create a 2D COF (COFETTA-TPAL) with dual pore diameters [132]. The nanosheet COFETTA-TPAL possessed a super crystalline conjugated structure having double pore sizes of 3.06 nm and 0.87 nm. Interestingly, the MP-11 and GOx paired right with COF’s big and small pores, allowing for pore diffusion. Furthermore, hydrogen bonds produced a b/w N atom of COFETTA-TPAL, and the –COOH of GOx and MP-11 may aid in enzyme immobilization [123].

Protein can completely or partially block the pore, as well as the fact that transport b/w reagents and products is limited; the fabrication of porous COFs is considered to truncate diffused path lengths to overcome these limits. Sun et al. demonstrated that porous COFs (hexagonal and triangular pores) can be used to encapsulate enzymes. The big hexagonal pores were loaded with enzymes, while the triangular pores allowed reagents and products to freely move in and out [132]. In the context of activity and chemical resistance to by-products, enzymes that fenced in COFs with double pores surpassed those that fenced in COFs with regular porosity [133]. Following that, a similar group has published the spatial configuration of enzymes in confined pore contexts and their effects on the degree of freedom of the hosted enzymes and subsequent catalytic activity [134]. They created three isoreticular COFs with varying hydrophilicity and studied and determined the relationship between the pores’ chemical environment, changes in the degree of freedom of the hosted enzymes, and the ultimate catalytic activity of these immobilized enzymes. With the rise in hydrophilicity of COFs, it was shown that lysozyme became more confined and had less activity. The immobilized enzyme’s reactivity changes in response to various host–guest interactions. This research enabled us to predict the function of biocomposites that are unknown. Interestingly, Chen and colleagues recently developed a vicarious drafting technique to create hollow COF capsules for encapsulating the enzymes and other biological molecules [135]. Enzyme@MOF was first created in digestible MOFs by in situ encapsulation to shield enzymes and aid as a template for the development of the enzyme @MOF@COF core–shell structure. After that, the MOF core was scraped away, allowing enzymes to be released into the hollow COF capsule. This method was discovered to work with a variety of MOF templates, such as ZIF-90, ZIF-8, and ZPF-2, and COF capsules, such as COF-42-B and COF-43-B). This unique technique created a comfortable atmosphere for enzyme molecules to eject, increased conformational freedom of enzymes, increased mass transfer and safety from the outer atmosphere, and ultimately increased the activity of enzyme. To enhance bioreactor performance, the COF shell could be adjusted finely. Furthermore, many enzymes could have been encased and trickled synergistically in COF capsules [123].

#### 4.4.9. Biocatalysis

**Figure 11:**
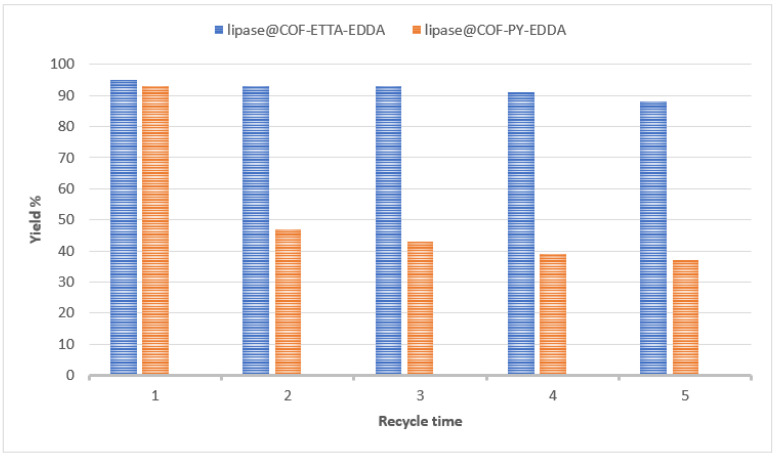
Recycling capability of COF-PY-EDDA and COF-ETTA-EDDA.

In recent years, biocatalysts have received a lot of interest as a sustainable and eco-friendly approach for the large-scale manufacturing of chemicals. However, their practical execution is frequently hindered by their worse ability to be recycled and long working stability [136]. Porous materials, such as zeolites, mesoporous silica, celite, COF, and MOF, are being studied as molecular flasks for storing enzymes. Sun et al. recently published a report in which they used 2D COF (imine-based) as a biomolecular flask for the immobilization of enzymes [125]. As a first precursor material, the authors used COF-OMe, which is made from 1,3,5-tris(4-aminophenyl)-benzene and dimethoxy terephthaldehyde condensation. COF–mesoporous OMe’s channel, which is 1D, with a pore size of 3.3 nm, can successfully encapsulate amano lipase PS with the same small length while maintaining its activity. The enzyme COF biocomposite (COF–OMe–PS) surpasses enzymes that are free and other biocomposites that are made from different porous materials, such as metalorganic framework and mesoporous silica, in catalytic activities, according to the authors. They also showed that the function of such host-immobilized enzymes may be regulated by altering the inner pore wall functions of host COFs while preserving the very same reticular chemistry [133]. The same group has described an exciting technique for using porous 2D COF as a host material for immobilizing enzymes. It improves the activity of enzymes, robustness, and ability of the catalyst to be recycled. COF–ETTA–EDDA, a star-shaped 2D COF with dual-pores created by the scientists, contains huge hexagonal mesopores at the center with 3.98 nm diameter and are walled by triangular micropores. When compared to the single-pore analogue, COF–PY–EDDA, the dual-pore 2D COFs have much a higher enzymatic activity and recycling capability (Figure 11) in the transesterification reaction, which is catalyzed by enzymes [136]. The improved efficiency of porous 2D COF is thought to be due to byproduct-active diffusion via tiny pores, which prevents the blockage of active sites of the enzyme, while big pores are involved in the conservation of enzymes after catalytic cycles. The adsorption efficiency of guest molecules may be greatly influenced by modifying the pore wall of the host COF. Lohse et al. recently synthesized B-ketoenamine-based 2D COFs. Samui et al. investigated a 2D COF which is an amine-based COF for the preservation of a-amylase, which is an industrially important enzyme [126].

#### 4.4.10. Antibacterial COFs

Three guanidinium halide-based ionic covalent organic nanosheets, which can be self-exfoliated and possess antibacterial properties, have been produced by Mitra et al. The concept of self-exfoliation was aided by molecular dynamics (MD) modeling. Self-exfoliation and antibacterial efficacy against gram-positive and gram-negative bacteria are both dependent on an inherent ionic guanidinium unit. A mixed-matrix membrane using such iCONs can be helpful for antibacterial coatings with potential medical profits [137].

Mal et al. produced 2D-iCONs based on propidium iodide that effectively block bacterial growth after exfoliation, whereas chemical-mediated restacking of the nanosheets results in loss of antimicrobial effect, which may be recreated by the removal of a chemical which controls bacterial growth [138].

COFs-Trif-Benz and COF-SDU1 are covalent organic framework (COF) materials and were discovered to be efficient type-II photosensitizers for the inactivation of bacteria photodynamically. COFs–Trif–Benz and COF-SDU1 are made from tri-(4-formacylphenoxy)-1,3,5-triazine (Trif) and benzidine or p-phenylenediamine in a simple solvothermal synthesis with good yield. Both Gram-positive *Staphylococcus aureus (S. aureus*) and Gram-negative *Escherichia coli* O86:B7 (*E. coli* O86) bacteria were investigated in the photocatalytic antibacterial assay [139]. After 60–90 min of irradiation, two materials may destroy more than 90% of bacteria at 100 μg mL^−1^ concentrations. Both COFs are photosensitizers. The antibacterial properties of COFs–Trif–Benz and COF-SDU1 were discovered to generate reactive oxygen species (ROS) via the transfer of energy to oxygen (^3^O_2_), resulting in highly reactive singlet oxygen (^1^O_2_) [139].

**Figure 12:**
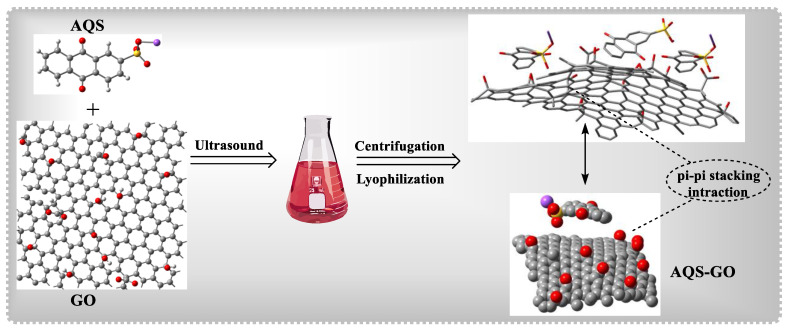
Loading of AQS onto GO, resulting in the AQS-GO nanocomposite through π–π interactions. “Reprinted from, Enhanced photo-induced antibacterial application of graphene oxide modified by sodium anthraquinone-2-sulfonate under visible light by Zhang, L.; Chen, P.; Xu, Y.; Nie, W.; Zhou, Y. Applied Catalysis B: Environmental 2020, 265, 118572. Copyright (2022), with permission from Elsevier” [142].

In a recent study, Chakraborty and colleagues examined mechanisms for physical cutting and oxidative stress of both GO and reduced GO (rGO) [140]. The difference in bactericidal efficiency b/w Gram-positive and Gram-negative bacteria was discovered and depends on size, shape, and kind. When compared to Gram-negative *P. aeruginosa,* Gram-positive *S. aureus* was found to be more susceptible to GO. The oxygen groups in GO allow bacterial cells to wrap around each other and prevent nutrients from entering. Furthermore, oxidative stress is formed on the cell membrane, causing it to destabilize and disintegrate, resulting in cytoplasmic fluid leakage. The importance of GO nanosheets for MDR bacterial treatment was recently demonstrated [141]. *E. coli, K. pneumoniae, S. aureus, P. aeruginosa, P. mirabilis*, and *S. marcescens* are MDR bacterial strains. Determined to increase GO’s ROS production, Zhou and colleagues used p–p interactions to load the phototherapeutic sodium anthraquinone-2-sulfonate (AQS) onto GO, resulting in the AQS–GO nanocomposite (Figure 12) [142]. When compared to GO under radiation, AQS–GO showed light-based inhibitory action against E. coli, with evident cell damage (Figure 10). Quaternary ammonium salts (QASs) are well-known as antibacterial agents [143,144]. Ye and colleagues created a GO nanocomposite with dodecyl dimethyl benzyl ammonium chloride. The QAS was employed as a bacterial-targeting component and an antibacterial reagent [145]. Both *E. coli* and *S. aureus* were resistant to systems for periods. Hao and colleagues recently described the use of PEG-functionalized GO in the fabrication of stable aqueous AgNP nanocomposites. AgNPs–GO–PEG had strong aqueous durability, minimal cytotoxicity, and protracted antibacterial activity (B95 %) against *E. coli* and *S. aureus*, while non-PEGylated GO with Ag aggregated irreversibly in aqueous solution [146]. Qu and colleagues in 2020 described the creation of a MOF@COF hybrid with nanozyme properties for the treatment of bacteria. COFs were used for the first time in that study to modulate the catalytic and therapeutic performance of MOFs, resulting in effective *E. coli and S. aureus* treatment [147,148].

Wang et al. used the Schiff base reaction to covalently connect two materials, MXenes and COFs, and attached Ag nanoparticles (NPs) to create a Ti_3_C_2_/TpPa-1/Ag composite material with efficient bactericidal properties. The material’s stability was considerably improved by the covalent connection between MXene and COF. Antibacterial activity of Ti_3_C_2_/TpPa-1/Ag composite against *S. aureus* and *P. aeruginosa* was outstanding [149]. The fluorescence spectra of Ti_3_C_2_/TpPa-1/Ag demonstrated that electron transfer channels established between the ternary materials might improve carrier separation efficiency and extend the life of photogenerated carriers. Calculations using density functional theory revealed that synergistic catalytic action of Ag and Ti_3_C_2_ may significantly decrease the work function along the interface, and a built-in electric field in among layers promotes rapid movement of the carrier, leading to improved catalytic performance [149].

#### 4.4.11. Wound Healing

Bacterial infections and inflammations usually accompany skin wounds, resulting in prolonged wound healing, which remains a major therapeutic challenge. As a result, it is essential to produce wound dressings that restrict bacterial infections and speed up wound healing [150]. The produced ENR–FMCOF–TPU fibrous membrane displayed excellent physicochemical and biological characteristics, including consistent and steady structure, adequate hydrophobicity, strong water uptake efficiency, and amazing biocompatibility, demonstrating ideal wound dressing behavior. Furthermore, the ENR–FM–COF–TPU membrane provided constant enrofloxacin and flunixin meglumine drug release and demonstrated potent antibacterial properties against *Staphylococcus aureus* and *Escherichia coli* with a 99% inhibitory efficiency for 50 h. Wound healing therapy effectiveness was investigated using a full-thickness skin defect model in mice. It was indicated that by downregulating inflammatory cytokines (IL-1 and TNF-) and enhancing the expression of growth factors, the ENR–FM–COF–TPU membrane might greatly speed up and promote wound healing (VEGF and EGF). The ENR–FM–COF–TPU membrane offers considerable possibilities in wound healing applications due to its exceptional characteristics [151].

The condensation process and the Schiff base reaction are used to make a curcumin-loaded COF (CUR@COF), which is then electrospun onto polycaprolactone (PCL) nanofibrous membranes (CUR@COF/PCL NFMs) to create a pH-triggered drug release platform for wound dressing. CUR@COF has 27.68% CUR loading capacity, and CUR@ COF/PCL NFMs have better thermal durability, mechanical characteristics, biocompatibility, and antibacterial and antioxidant activities. More crucially, CUR@COF-based membranes exhibit a pH-responsive CUR release profile through protonation under acidic conditions, implying that an acidic extracellular milieu promotes CUR release from membranes. CUR@COF/PCL NFMs can speed up wound healing and skin regeneration by reducing the expression of inflammatory factors (TNF-a) and increasing the expression of angiogenesis according to histopathological examination and immunofluorescence labeling of an in vivo skin defect model (VEGF) [152].

As a result, a new TP-Por CON@BNN6-integrated heterojunction for destroying Gram-negative bacteria *Escherichia coli* and Gram-positive bacteria *Staphylococcus aureus* in vitro was successfully manufactured. TP-Por CON@BNN6 possesses promising biocompatibility and biodegradability, low phototoxicity, anti-inflammatory capabilities, and outstanding wound healing abilities in mice in vivo. The TP-Por CON@BNN6-integrated heterojunction with multifunctional capabilities appears to be a promising technique for COF-based gaseous therapy and microorganism-infected chronic wound healing [153].

#### 4.4.12. Anti-Viral

The latest COVID-19 pandemic has sparked global health worries. To combat the COVID-19 pandemic, stopping severe acute respiratory syndrome coronavirus-2 (SARS-CoV-2) activity in the body is a promising strategy [154]. One of the protective strategies is to limit the binding process between the human cell receptor ACE2 and the coronavirus spike protein. By using a computational technique, the effect of the deformation of the spike protein structure produced by covalent organic frameworks (COFs) by decreasing interactions between ACE2 and spike protein was investigated. [155].

**Figure 13:**
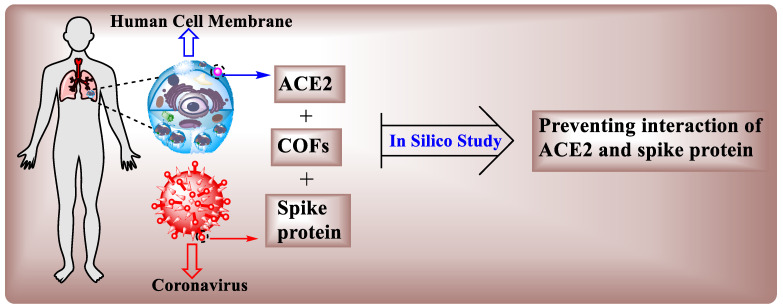
Schematic representation of ACE2 and COF preventing interaction of spike protein with ACE2. “Reprinted from, Atomistic insight into 2D COFs as antiviral agents against SARS-CoV-2. By Jahromi, A.M.; Solhjoo, A.; Ghasemi, M.; Khedri, M.; Maleki, R.; Tayebi, L. Materials Chemistry and Physics 2022, 276, 125382, Copyright (2022), with permission from Elsevier” [156].

A molecular dynamics simulation is used to provide an atomic characterization of interconnections between ACE2 and the spike protein in this case [157]. First, we looked at the interactions of three distinct COFs with the spike protein, including COF-78, DAAQ-TFP, and COF–OEt, by assessing bond energies and structural alterations in the spike protein. First, interactions among three distinct COFs with the spike protein, including COF-78, DAAQ-TFP, and COF–OEt, were examined by assessing bond energies and structural alterations in the spike protein. The distorted spike protein’s intermolecular interactions with ACE2 were then examined to better understand the protein’s fusion after deformation. According to the findings, while all the COFs deformed the spike protein effectively, COF-78 performed well in terms of preventing spike protein–ACE2 interconnections by altering the protein’s molecular structure [156]. Interestingly, COF-78’s interaction in the study of the deformed spike protein with ACE2 revealed that its interactions had the lowest absolute energy, as well as few hydrogen bonds, indicating that the protein’s compaction was lower than that of the other deformed proteins. Furthermore, COF-78’s positive performance was confirmed by its high contact area with an aqueous solution and significant variations during the simulation duration (Figure 13). New materials and COVID-19 prevention methods were introduced that might be employed for surface disinfection in face masks [156].

## 5. Conclusions and Future Prospects 

COFs have a porous framework and are more versatile than any other porous materials. The development of COFs is still in its early stages. The unique physical and chemical properties make them more reliable to use, especially regarding high chemical and thermal stabilities. Strong covalent bonding is present between the linkers; hence, COFs can be modified easily. Organic linkers such as FGs undergo polymerization to form COFs. COF-derived materials can be used in different applications, including environmental remediation and catalysis.

The unique characteristics of COFs are their extremely porous nature and stability. These characteristics make COFs more powerful tools for environmental remediations. Due to their porous nature, the adsorption, filtration, and separation of different components through COFs is made possible. The large porous size allows bulky organic molecules to pass through them. COFs do not contain metal atoms/ions in them, which is another powerful feature of COFs to be used as environmental remediators. COFs are ecofriendly in many ways. For the removal of contaminants in water, COFs are used for degradation. With the help of luminescent COFs, even low concentrations of contaminants can be detected.

The use of COFs as biomaterials is still in its early stages, it is essential to determine specific areas where COFs could be useful. COFs, like polymeric biomaterials, can be designed and functionalized in a variety of ways. Medicinal components can be loaded into COFs through built-in and tunable porosity channels. To accomplish functional roles, the drug entrapment amount and release rate of COFs can be modified within a specific range by systematically adjusting pore size and polarity. COFs can be employed to enhance the photothermal effect of nanomaterials or as transporters for photothermal agents in photothermal therapy. In contrast to a closed porous structure, which impedes heat transfer, COFs can rapidly transport temperature. COFs possess high load capacity because of their systematic pore structure and high specific surface area. COFs are employed to transport drugs to the target in drug chemotherapy. COFs with a wide surface area and porosity, high electrical conductivity, and novel interactions with bioactive compounds could diagnose (biosensing and bioimaging) cancer.

COFs can be used for metal catalytic applications (heterogeneous catalysis) as they do not contain metal atoms/ions in them. COFs have high thermal and chemical stabilities with adjustable active sites. These properties make COFs ideal porous catalysts. COFs such as COFs with reactive metals, COFs with molecular catalysts, COFs with reactive linkages, and COFs with reactive pendant groups can be made either in post-synthetic, in situ or bottom-up methods. COFs have been used for various catalytic processes like photocatalysis, chemical catalysis, and electrocatalysis for almost a decade. Two-dimensional and three-dimensional COF- based catalysts with unique structural and chemical features have been designed to catalyze different reactions, such as redox reactions, organic coupling reactions, degradation, and addition reactions. COFs showed better catalytic activities than other catalysts. COF base photocatalysts have been designed with photoactive linkages by installing photoactive metals in the porous confinement of COF. The photocatalytic COFs are used for organic redox reactions, CO_2_ reduction, water splitting, radical formation, and organic transformation reactions. COF-based catalysts possess better structural activity relationship understanding, good confinement effect, size sieving and mass transfer, and better access of substrate concentration and catalytic site due to their high surface area. With COF-based catalysts, catalytic reactions can proceed through different mediums. Catalytic sites can be easily installed, and guest species can be incorporated easily due to manageable pore space.

Despite all the efforts made for the development of COFs, the development is still in its early stages. There is still more to be developed; for example, 3D COFs in comparison to 2D variants are less explored. The development of 3D COFs is extensively needed for photocatalytic and electrocatalytic applications.

The following challenges must be addressed: Facile methods should be explored for COF synthesis. The assembly of COF-based functional materials should be investigated, and a relationship should be established between structure and performance. Cost effective linkers should be selected so that further advancement of COFs can be done. COFs should be assessed against environment hazards. Recycling and reuse of COF-based materials should be addressed for more advancements in COFs.

## Data Availability

Not applicable.
