# Correction: Bukhari et al. Covalent Organic Frameworks (COFs) as Multi-Target Multifunctional Frameworks. *Polymers* 2023, *15,* 267

**DOI:** 10.3390/polym17111443

**Published:** 2025-05-23

**Authors:** Syed Nasir Abbas Bukhari, Naveed Ahmed, Muhammad Wahab Amjad, Muhammad Ajaz Hussain, Mervat A. Elsherif, Hasan Ejaz, Nasser H. Alotaibi

**Affiliations:** 1 Department of Pharmaceutical Chemistry, College of Pharmacy, Jouf University, Sakaka 72388, Saudi Arabia; 2 Department of Pharmaceutics, College of Pharmacy, Jouf University, Sakaka 72388, Saudi Arabia; 3 Center for Ultrasound Molecular Imaging and Therapeutics, Pittsburgh Heart, Lung, Blood and Vascular Medicine Institute, University of Pittsburgh, Pittsburgh, PA 15213, USA; 4 Centre for Organic Chemistry, School of Chemistry, University of the Punjab, Lahore 54590, Pakistan; 5 Chemistry Department, College of Science, Jouf University, Sakaka 72388, Saudi Arabia; 6 Department of Clinical Laboratory Sciences, College of Applied Medical Sciences, Jouf University, Sakaka 72388, Saudi Arabia; 7 Department of Clinical Pharmacy, College of Pharmacy, Jouf University, Sakaka 72388, Saudi Arabia

## Error in Citation

In the original publication [1] there was an error in “Reference 69”. The citation has now been corrected and should read as follows:

69 Ahmed, I.; Jhung, S.H. Covalent organic framework-based materials: Synthesis, modification, and application in environmental remediation. *Coord. Chem. Rev.* **2021**, *441*, 213989. https://doi.org/10.1016/j.ccr.2021.213989.

The authors state that the scientific conclusions are unaffected. This correction was approved by the Academic Editor. The original publication has also been updated.
